# Collectanea

**Published:** 1843-12

**Authors:** 


### Collectanea.

*The Tartar on the Teeth.**—A soft substance, of a whitish or yellowish color, is habitually deposited upon the teeth, and sometimes becomes firmly fixed to them. This substance may accumulate in greater quantities, and growing firmer by degrees may form the hard and dry concretion known under the name of tartar. It increases in bulk by the fresh layers deposited on its surface. According to an analysis made by Vauquelin and Laugier, tartar consists of sixty-six parts of phosphate of lime, nine of carbonate of lime, fourteen of animal matter, (of a yellowish white, different from the gelatine of bones,) and three parts of oxide of iron and phosphate of magnesia. Other chemists have found the proportions different; sometimes the phosphate of lime was more abundant, and sometimes the animal matter (or mucus.)

Authors have been much occupied with the manner in which this substance is produced. Is it a secretion, as some have written? Is it a deposit of the earthy salts contained in the saliva, and precipitated by a chemical agent, as medical books have reported for ages? Is it an earthy exhalation from the capillaries of the blood, to which the mucous membrane of diseased gums is prone?

Not one of these hypothesis has been proved; not one has the sanction of direct experience. Moreover, they are all sufficiently refuted by the following investigations into the composition of tartar.

It results from the experiments of M. Mandl that tartar is nothing but a deposit of the skeletons of dead infusoria, agglutinated by dried mucus; nearly as certain earths, according to the researches of M. Ehrenberg, are composed almost entirely of fossil infusoria.

In fact, if we take some of the mucous matter which is accumulated upon and between the teeth, and dilute it with a little distilled water previously warmed, we shall immediately perceive a host of infusoria, which move about with great liveliness. Their size varies from 1-500 to several hundredths of a millimetre; and their shape is the same as that of the infusoria described by authors under the name of *vibriones*.

The presence of infusoria in mucus was pointed out by Leuwenhœk; but M. Mandl sets forth in all their details, the shape, liveliness, and other qualities of these infusoria.

These animals also exist in great quantity in patients who have been

* Read by M. Mandl at the Academy of Science.

several days on low diet. They also constitute the greatest part of the mucous coating of the tongue in persons whose digestion is disordered. (According to an analysis of M. Denys, the chemical characters of this coating agree with those of tartar.)

After having ascertained the presence of infusoria in the mucus of the mouth, M. Mandl tried to find out whether these animals assist in forming the tartar also. For this purpose he softened a particle of tartar in a drop of water for twenty or thirty minutes, and after compressing it between the two pieces of glass, he distinctly saw that the tartar was composed of dead vibriones, of different sizes, but generally measuring several hundredths of a millimetre, united by an organic substance (dried mucus) the quantity of which is variable. The tartar is often almost entirely composed of these vibriones.

Hence it follows that these vibriones are provided with a shell, or inorganic skeleton [*squelette inorganique,*] since tartar is found consisting entirely of these vibriones.

This shows, too, why cleanliness, and the use of tonic or alcoholic fluids, prevent the formation of tartar by preventing the production of the infusoria.

To recapitulate, it appears:

1. That there is a great number of vibriones in the mucus which accumulates around and between the teeth.

2. That tartar arises from an accumulation of dead vibriones, and consequently cannot be considered either as a calcareous substance deposited by the saliva, or as a peculiar secretion.

This discovery of the composition of tartar is as new with reference to Leuwenhœk's observation, as the researches of Ehrenberg touching the composition of diluvial soils were new in reference to the well-known fact of the existence of infusoria in water.—*Gaz. Medicale, but now from Boston Med. and Surg. Jour.*

*Continental Treatment of Neuralgia.*—Dr. Schleiser, of Peitz, has prescribed, with success, to patients with abdominal neuralgia, but whose circumstances would not permit of their visiting a watering-place, the use of an artificial mineral water, resembling that of Eger, in Bohemia, and made as follows:—*R. Filtered spring-water,* a pint; *diluted sulphuric acid,* two drachms and a half; *hydrochloric acid,* twenty drops. Mix, and add *bicarbonate of soda,* forty-five grains. The bottles are then to be sealed up without delay, and kept cool; one or two pints may be drunk daily. In hepatic neuralgia, Dr. Schleiser depends much on the effects of belladonna; in cases where great irritability of the stomach is present, he finds nitrate of silver suitable, combined with morphia.—*Rust's Magazine.*

Morphia has been an ordinary remedy for neuralgia, the cure of which it may, in certain cases, effect; but a French practitioner, M. Rougier, has advised the adoption of an ingenious method, which he says will prove the completeness and permanance of the cure. After the apparent removal of the disease by the morphia, he administers successive small doses of strychnia, gradually increasing the amount of the doses and abridging the intervals between them. Now, if the cure have been complete, the tremors and other characteristic effects of the strychnia go on diminishing in intensity from the first, notwithstanding the increasing strength and frequency of the doses; but if otherwise a contrary result happens, and the effects of the strychnia increase in intensity.—*L'Experience.—London Lancet.*

*Neuralgia from Sympathy with a Diseased Tooth.*—A woman, twenty-seven years of age, was accustomed to be awakened at night with a sharp and lancinating pain—in fact, tic douloureux—in the left cheek, which extended to the temple, to behind the ear, and along the anterior border of the trapezius muscle. An hour or two in the morning, during which she slept, appeared to be the sole respite she had from pain. All kinds of rubefacients and narcotics were employed without any more than momentary benefit, when it was perceived that the second molar tooth was carious, and after some delay, it was extracted, when all the pains immediately ceased. Only the enamel of the tooth had been decayed; its roots were sound.—*Gazette des Hopitaux.—London Lancet.*

*Dental Ingenuity.*—By turning to some of the back volumes of this Journal, an account may be found of the case of Jeremiah Driscoll, of Warren, R. I., who was injured, in the South Pacific Ocean, by a whale, Nov. 7th, 1836. It is only necessary to repeat the fact, that the end of an oar was driven into his mouth, in such a manner as to knock off the whole anterior part of the alveolar arch of the upper jaw, teeth and all; the roof of the mouth was broken through, and the nasal cavity freely admitted the tongue into it—the bones having subsequently come away. This is only a general outline of a terrible wound, from which Mr. Driscoll finally recovered, but greatly disfigured, and wholly unable to masticate food. By the skill of a Boston dentist, Dr. Harwood, now residing at Machias, Me., the deformity was measurably overcome, and the ability to subsist on solid aliment restored to him by a complete set of teeth.

Four years, however, have considerably modified the shape of the mutilated jaw, so that there was considerable difficutly in maintaining the palate plate in its proper position. Consequently, articulation as well as manducation was beginning to be an exceedingly troublesome process, to say nothing of the distortion of features that must necessarily ensue from any imperfection in the mechanism of such an extensive artificial surface as he had been wearing. Under these circumstances, he has again recently visited Boston for assistance. Dr. Joshua Tucker, whose ingenuity has often been put in requisition in cases equally perplexing, succeeded in admirably fitting a new palatine and alveolar arch, bearing a highly finished set of molar and incisor teeth, which have as perfectly restored Mr. Driscoll as it is possible for art to do. No one would suspect, without a minute examination, that so much of the man was artificial.

These triumphs of art, of such immense importance to those who have been unfortunately maimed, should be extensively circulated by the press, that all sufferers may avail themselves of the advantages accruing from the modern discoveries and improvements in mechanical dentistry.

*Bost. Med. & Sur. Jour.*

*Advantage of System in Medical Inquiries.*—"I advise a systematic arrangement in each case; observe the state of the pulse and skin; feel the head (in insane patients) whether it is hot all over, or in one part only; whether the extremities are cold; whether the tongue is loaded and dry; whether the bowels are open, the urine free, and if the patient be a female, whether the catamenia are regular. Next observe the breathing and the action of the heart, pass your hand over the right hypochondrium, and feel whether the liver be enlarged, or whether the abdomen be distended with flatus, and whether there be tenderness about the precordia. Examine also the beating of the carotids and the temporal arteries."—*Dr. Sutherland.*

*Illustrations and Sketches of Medical Quackery.*—Walpole says that acute and sensible people are often the most easily deceived by quacks. A deceit, of which it may be said—"It is impossible for any one to dare it," always succeeds.

If the imposture required any ingenuity to detect it, there might be some hope for mankind; but it actually lies concealed in its *very obviousness.* At the same time it must be owned, that, in some cases, no little degree of firmness is required to resist the importunity with which a nostrum is recommended. "I seriously declare," says Sir A. B. Faulkner,* "that I was myself pressed with no little earnestness, by a person not otherwise above par in credulity, trying to persuade me of the infallible powers—of what?—Ye shades of Hippocrates and Æsculapius—what?—actually and seriously, a decoction of flint stones!!! The prescription was grave and methodical. The flints were to be boiled, and the supernatant liquor poured off for use. The lady who advised this precious physic, would do so on the best authority; and not of one, but of many persons of her acquaintance, upon whose word she could place the most implicit reliance."

The charm is in the *mystery,* in all these cases. *"Minus credunt quæ ad suam salutem pertinent, si intelligunt,"* says Pliny. Credulity is indigenous in no particular climate. "In Chili," says Zimmerman, "the physicians

* Visit to Paris.

blow around the beds of their patients, to drive away disease; and, as the people in that country believe that physic consists wholly in their wind, their doctors would take it very ill of any person who should attempt to make the method of cure more difficult." They think they know enough when they know how to blow; which, translated into common language, means, "raising the wind."

Lord Bacon says, "That the impostor frequently triumphs at the bed-side of the sick, when true merit is affronted and dishonored; the people have always considered a quack, or an old woman, as the rivals of true physicians. Hence it is that every physician, who has not greatness of soul enough not to forget himself, feels no difficulty in saying with Solomon, *'if it is with me as with the madman, why should I wish to appear wiser than he is?'* "

> "The world is generally averse
> To all the truths it sees and hears;
> But swallows nonsense and a lie,
> With greediness and gluttony."
>
> BUTLER.

*Physic and Physicians.—Med. News.*

---

*Climate and Characteristics of Nice.*—Nice, a city with nearly thirty-four thousand inhabitants, is situated on the Mediterranean, just within the confines of Italy, on a promontory, proceeding from a range of the maritime Alps, in lat. 43° 41′ N., and long. 7° 16′ E. Its climate is supposed to be the mildest on the north coast of the Mediterranean, owing to the gradually rising lines of hills which shelter it from the N. E. round to the W., leaving it open to winds only on the S. and S. E. From observations, continued from 1806 to 1838, the mean height of the barometer has been found to be 27.11, the range being only between 26.11 and 28.8. Out of 36,135 observations of the thermometer, it was only once seen to rise so high as 92° 5′ Fah., the lowest point having been 15° Fah. The maximum of the hygrometer was 100°, the minimum 17°, the mean 59°. The *tramontane* north wind seldom descends into the lower plain of Nice, but sweeps directly from the mountains down to the sea; the east wind is dry and cutting, the south relaxing, the S. S. W. wind, or *lebec,* is very prejudicial to health. Dew is abundant, but mists are extremely rare. The soil of the environs is an aggregate of chalk, clay, and sand, and of much fertility. The inhabitants are generally of low stature, with a muscular but not fleshy frame, and a pale complexion generally. It is remarked, that where oil is much used for food, the complexion is pale, while a diet of milk is attended by a rosy and fresh color.

The lower class of females at Nice become marriageable at from twelve to thirteen years of age, and mothers of thirteen years are not uncommon. Young ladies marry at from fifteen to twenty, young men at from eighteen to twenty- four. Child-bearing is said to cease at from forty-three to forty-five years of age. The average length of life is thirty-one years.

The most prevalent diseases are those of an inflammatory kind—pneumonia, pleurisy, catarrh, &c., occasioned by sudden alternations of temperature, variable winds, and an atmosphere often charged with irritating matter. Pulmonary complaints have latterly been more numerous among the population (probably owing to Nice having become more than formerly a place of resort for consumptive patients.) In the town and immediate district of Nice, from 1828 to 1837 inclusive, there were born 10,968 persons; the deaths in the same period amounted to 9,163. In the south of the province of Nice, many mothers are found with eighteen and twenty children; twelve are a frequent occurrence, and from six to eight are quite common; but the average number of children to a marriage is estimated at from four to five. The number of marriages in the province annually is computed at from ten to twelve in every one thousand of the inhabitants.—***Sir J. P. Boileau, in Statist. Jour. for August,*** 1843.—***London Lancet.***

---

*Theory of Animal Heat.*—By J. M. Winn, M. D., Truro, England.—About three years since, whilst making a few experiments with caoutchouc, I was forcibly struck with the property it possesses of evolving heat when suddenly stretched, and was led, at the time, to infer the probability of other bodies being similarly endowed. The elastic coat of arteries, especially, from the mechanical resemblance it bears to caoutchouc, appeared to be one of the substances most likely to exhibit this calefactory principle; and in the event of this being the case, it would not be unreasonable to conclude that the incessant contractions and dilatations of the arteries during life, must form an efficient source of animal heat.

During the past week I was induced to resume the subject afresh; and upon making an experiment with part of the aorta of a bullock, I felt much gratification in being able to verify my previous conjecture. The experiment was performed in the following manner:—Having cut off a circular portion of the descending arch of the aorta, about an inch in length, I laid it open and carefully dissected out the elastic coat, and taking hold of it by each extremity, I pulled it to and fro, with a continuous jerking motion, (in imitation of the systole and diastole of the artery,) for the space of about a minute, when placing it on the bulb of a thermometer, I had the satisfaction to find that after it had remained two minutes the mercury had risen as many degrees. On removing the thermometer, the heat immediately began to diminish. To be certain that the heat did not arise from any other source than the one in question, I took the precaution of covering my fingers with a double layer of flannel, to prevent the communication of heat from the body; I also covered my mouth with a handkerchief, to guard against the warm breath affecting the thermometer, whilst watching the progress of the experiment. I may likewise state that the experiment was performed in a room without a fire, the temperature of the air at the time being 55°. There were several difficulties to contend with during the investigation, and it was not until after repeated trials that the experiment succeeded to my satisfaction. The chief impediment, I think, must have been owing to the moisture of the artery, which, by its evaporation, must have had a constant tendency to carry off the heat. Having, however, performed the experiment twice consecutively, in the same satisfactory manner, I think there can be but little doubt entertained as to its conclusiveness. My attention was often arrested, whilst conducting the experiments, by the striking mechanical analogies between caoutchouc and the elastic coat of the arteries. Like the former, the latter could be elongated to twice its ordinary length, and on withdrawing the tension would return to its usual dimensions, with considerable force and a snapping noise. I was also surprised to find, on slightly drying it, that it would erase black-lead pencil marks from paper, without leaving a stain. This latter circumstance is perhaps of trifling importance; it serves, however, to show that strong mechanical resemblance may exist between bodies widely differing in their chemical properties.—*Philos. Mag.—Bost. Med. & Sur. Jour.*

*Swallowing a five-franc piece—Its extraction from the œsophagus.*—At the end of 1838, a farmer in France consulted a medical practitioner for the consequences of his folly in swallowing for a wager, a five-franc piece, a coin about the size of an English crown. (We had thought that such absurd bets were confined to John Bull, but the French journals have lately recorded several instances showing that our neighbors are not disposed to be far behind us.) The man in question had suffered for the space of eight days from the presence of the coin in the œsophagus; he could swallow only milk, broth, and other liquids, and was on the high road to perish of hunger.* Pale and half dead with fright, he now applied to M. Monin, who reports the case. That gentleman, on passing an India-rubber sound into the œsophagus, ascertained that the coin was impacted there at about the junction of the upper two-thirds with the lower third of the tube; and he suspected, from being unable to pass his instrument beyond it, and from other signs, that the body was placed horizontally, so as nearly, if not wholly, to block up the passage. The membrane of the œsophagus was so closely contracted around the coin, that the elastic sounds of caoutchouc, whalebone, &c., at first used, had not force enough to compel the latter body to assume a more favorable position. In these embarrassing circumstances M. Monin caused some forceps to be made, the limbs of which were curved so to form the segment of a circle, and which opened sideways in the direction of the mouth, and *not vertically*,—a point strongly

* And of fatigue also, if dancing can fatigue a Frenchman. "For a practitioner to whom he had first applied recommended him to live on oil, and to *take to jumping*, to facilitate its entrance into the stomach, and for eight days he had religiously jumped as high as he could."

insisted on by M. Monin. The inside of each limb, at its extremity, was roughened with a file. This instrument, well oiled, was introduced with ease into the œsophagus of the patient, the left forefinger of the operator being placed over the root of the tongue and epiglottis. With a small degree of force, the instrument now obliged the coin to place itself in a vertical position, or with its rim upwards, and in a few moments it was seized by the forceps, and being firmly grasped, slow and gentle traction was employed. This was continued till the coin arrived in the pharynx, where, being arrested by the arch of the palate, it suddenly escaped from the hold of the operator. The pressure it now exercised over the glottis caused symptoms of imminent suffocation; but a sharp blow between the shoulders speedily caused the expulsion of the coin from the mouth.

The operator, who has a humorous way of reporting the case, says, "to make a bound, and dash after the object which had caused him so much terror and suffering, was the first impulse of the poor patient, who disappeared like a flash of lightning, *(partit comme une eclair,)* without paying the least attention to my recommendation of moderation in diet after his long starvation. I did not see him for several days afterwards, when he complained of nothing but a little soreness at the point where the piece of money had been impacted. He experienced no other ill effects from his imprudence.—*London Lancet.*

*Secret Remedies in France.*—The sale of secret remedies is prohibited in France, unless legally protected by patent, which is never granted without a report from the Royal Academy of Medicine, certifying the harmlessness of the remedy. This is not the only hinderance to the sale of those compounds. Our London warning, "Bill-stickers, beware!" is uttered with more authority by the French law; and it would appear that the Parisian quack is destroyed, like a parasitic plant, by removing him from the wall. This fact is thus exemplified: "A practitioner caused a placard to be affixed on the shop of a grocer in the Rue St. Denis, advertising a remedy against secret complaints. The grocer brought an action against the doctor, who was condemned in 2*l*. damages. The court cautioned Dr. M. not to affix placards on the walls lest he should incur renewed actions for damages."

If it were made illegal to post the walls thus, or to advertise them in the newspapers, quack medicines could not exist. Few empirical preparations could stand the silent system. Again, M. Boubee is a regularly educated *pharmacien,* rivalling M. Beral in the preparation of citrate of iron, and established at Auch. He contrived a syrup to banish gout from France, affixed his name to it, and in time sold it in several shops at Paris. But M. Boubee forgot to register the exact composition of his sedative solution. This at once turned the syrup into a secret remedy; and he was cited before "Le Tribunal" to answer for the advertisement and sale of secret remedies. Not appearing, he was recently condemned to ten months' imprisonment, and a fine of 24*l*.—*Ann. of Chem.*—*Med. News.*

### FRENCH MEDICAL SOCIETIES.

*Academy of Medicine.*—July 4th. (M. Dubois, president)—M. Colombat, of Isere, read a memoir giving an exposition of his system for the cure of stammering. According to him, stammering is purely a nervous affection, a functional disease, and is of two kinds, *choreic* and *tetanic*. Choreic stammering consists in a kind of chorea of the lips, and a succession of convulsive movements, more or less rapid, of the tongue, lower jaw, and all the muscles of articulation. The stammering called by M. Colombat, tetanic, is marked, as he says, by a kind of tatanic rigidity (*raideur,*) or a tonic spasm of all the respiratory muscles, but eminently those of the larnyx, pharynx, and base of the tongue. This kind of stammering is most perceptible in the guttural letters, to articulate which is always a painful effort, signalised by an explosive and hurried sound (*expiration anticipée*) and then an interval of silence before the finishing of a word; by immobility of the tongue, closure of the glottis, a sense of pressure within the chest, and a momentary impression of suffocation. Individuals of the more active temperament and nervous excitability generally suffer from choreic stammering; these speak rapidly and commonly without effort, but with the repetition of certain letters—b-b-b—f-f-f, &c. On the other hand, in the *guttro tetanic* species of stammering, persons speak slowly, and evidently with effort. Both these kinds, however, often affect an individual at the same time. We need not enter into M. Colombat's numerous subdivisions of the two kinds of stammering.

Among the curative means proposed by the author are the following:—Patients with the choreic kind are advised to employ the zygomatic muscles pretty constantly, and while speaking, to open the commissures of the lips, as in smiling. With the lips brought into the resulting position, labial stammering is next to impossible, and the tongue also, carried backwards, facilitates the pronunciation of other than the labial letters. For the tetanic kind, another exercise is proposed, bringing into play at once all the muscles concerned in the production of speech. It consists in drawing an inspiration, at the same time forcing backwards the tongue, so that its point should approach the velum palati, while the lips are extended as above detailed for the choreic kind. At other times, in lieu of stretching the lips transversely, the mouth is extended vertically and the jaws separated as widely as practicable for the pronunciation of syllables. At this time, also, the tongue is to be retracted in the mouth to a distance of two or three lines behind the teeth. The hurried nature of the respiratory acts in stammering of this kind, consequent on spasm of the chordæ vocales, &c., is thus brought under control. By throwing back the tongue into the pharynx, in the manner indicated, the opening of the glottis is widened at the same time that the chest is filled and emptied of air. Several accessory means are employed by M. Colombat with a similar view. He directs the attention of his patients eminently to the *rhythm* of the sentences they employ, which tends to withdraw their minds in a salutary manner from noticing their physical defects; he inculcates the use of certain syllabic exercises, and for choreic stammerers especially he has invented an instrument which he calls the *muthonome.* This instrument acts somewhat like Mælzel's metronome, but is constructed on another principle, and will go for several days without being wound up. By its means, M. Colombat limits the syllables to which his patients give utterance at first to 60 or 80 in a minute, which number he gradually increases to 160 or 180 in the same space of time. The foregoing are among the methods by which M. Colombat has, as he says, in a period of fifteen years, restored the free exercise of speech to 782 persons, of whom 643 were cured without relapse. In general, from a month to six weeks perseverance in his treatment has been sufficient to obtain a cure, though it may sometimes have to be prolonged for about three months. In the Academy of Sciences, at the sitting of July 3rd, a memoir on the same subject, by M. Jourdant, was read, in which it was advanced that stammering was for the most part a consequence of too rapid expiration, obliging the patient to respire several times to enable him to conclude a sentence. The suggested treatment was, accordingly, as follows:—The patient is to perform a natural inspiration, the course of which is to be suddenly arrested. A sentence is then to be begun, slowly pronounced, and continued till it, or a fixed portion of it, is finished. Then the air remaining in the chest is suffered to escape altogether, and, after a short pause, the same process is to be recommenced. The tongue is to be maintained in an easy and natural position, "which is that in which it lies, for instance, after having swallowed the saliva." The medium time occupied in cure, according to this method, is reported to be a month, the patient practising the method recommended for two hours daily.

At the sitting of July 10th, M. Boussingault made a favorable report of a method proposed by M. Sirey for the removal of fæcal stench. The agents used for this purpose by M. Sirey are powdered charcoal and sulphate of iron.

M. Valenciennes read a paper on verminous tumors found in the stomach of the horse, the worms inhabiting which tumors, are altogether different from those found in the large intestine. Out of twenty-five horses, the stomachs of which he examined after death, either alone or in conjunction with M. Rayer, he had found these entozoa in eleven, in one of which were two verminous tumors, and in another four. They were situated between the mucous and fibrous coats of the organ, with the cavity of which they communicate by from one to five openings, and through these the worms easily pass. The tumor is internally subdivided into cells by folds of membrane secreting a mucus, which sometimes concretes in such a manner as to give the tumor almost a scirrhous hardness. The worms lodged in these cavities are supposed to be the *spiroptera megastoma* of Gurlt; but M. Valenciennes considers that they ought to be classed between the spiropteræ and the ascarides. The males are nearly half an inch in length, by about 1-50th of an inch in thickness; the females are somewhat larger and stronger, varying in length from half an inch to an inch and a third, and nearly a millimetre, (1-26th of an inch) in breadth.—*London Lancet.*

*Results of Homœopathy.*—We regret to state that another of the Earl of Denbigh's family has fallen a victim to the theory of infinitesimal doses. His lordship's third son died, last week, from accumulation of mucus in the air passages, the result of whooping cough. An emetic is usually employed, in cases of this kind, to free the bronchia; but whether the homœopathic fractions of ipecacuanha produced this effect or not, we have not heard.

*Prov. Med. Jour.—Western Lancet.*

*How to make Leeches bite.*—The leech, which it is intended to apply, is to be thrown into a saucer containing fresh beer, and is to be left there till it begins to be quite lively. When it has moved about in the vessel for a few moments, it is to be quickly taken out and applied. This method will rarely disappoint expectation, and even dull leeches, and those which have been used not long before, will do their duty. It will be seen with astonishment how quickly they bite.—*Med. Ex.—from Weitenweber's Beitr., and Schmidt's Jahrb.—Western Lancet.*

*Operation for Cataract.*—In a communication, signed "A Subscriber," we are informed that "Dr. Thos. H. Roe, of Newark, has lately performed this beautiful and skilful operation on James Green, of Muskingum county, a young man 28 years of age, who had been stone-blind from his birth." No unfavorable symptoms, we are informed, occurred, and the operation was successful. The patient had some difficulty in exercising his new sense, so as to judge correctly of distance and forms, and colors; though he acquired a knowledge of the latter sooner than of the former.—*Western Lancet.*

